# P-970. A Public Health Approach to Antimicrobial Stewardship in Long-Term Care Facilities: Benchmarking Antibiotic Prescribing in Massachusetts

**DOI:** 10.1093/ofid/ofaf695.1169

**Published:** 2026-01-11

**Authors:** Kap Sum Foong, Leslie Fowle, Amanda Slider, Maureen Campion, Majd Alsoubani, Jessica Leaf, Christina Brandeburg, Ashley Iannone, Melissa Cumming, Gabriela Andujar Vazquez, Shira Doron

**Affiliations:** Tuft Medical Center, Tufts University School of Medicine, Boston, MA; Massachusetts Department of Public Health, Boston, Massachusetts; Massachusetts Department of Public Health, Boston, Massachusetts; Tufts Medical Center, Boston, Massachusetts; Tufts Medical Center, Boston, Massachusetts; Massachusetts Department of Public Health, Boston, Massachusetts; Massachusetts Department of Public Health, Boston, Massachusetts; Massachusetts Department of Public Health, Boston, Massachusetts; Massachusetts Department of Public Health, Boston, Massachusetts; Tufts Medical Center, Boston, Massachusetts; Tufts Medical Center, Boston, Massachusetts

## Abstract

**Background:**

Antimicrobial stewardship in long-term care (LTC) facilities is crucial to curb antimicrobial resistance and reduce inappropriate antibiotic use. Yet, data-driven interventions remain underutilized in this setting. To address this gap, the Massachusetts Department of Public Health launched a voluntary Antibiotic Start (AS) benchmarking program in 2018, along with additional antimicrobial stewardship initiatives such as LTC office hours webinars and online educational materials. This study evaluates the impact of these interventions on antibiotic prescribing in LTC facilities.

**Figure 1.**
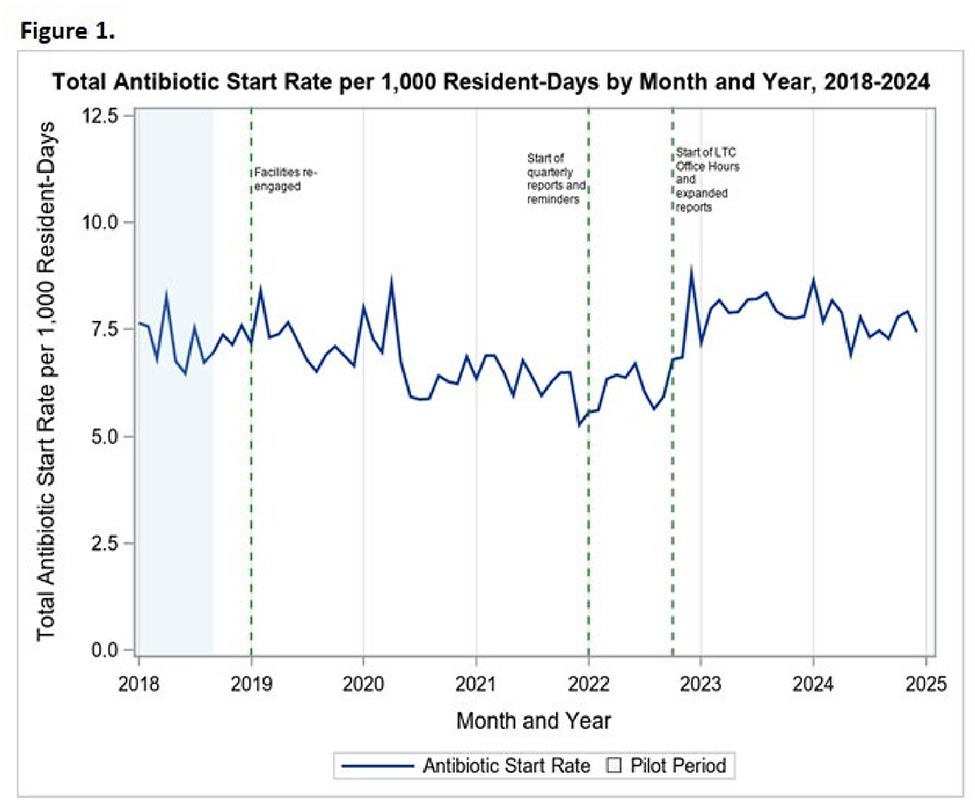
Total antibiotic start rate per 1,000 resident days by month and year, 2018-2024

**Figure 2.**
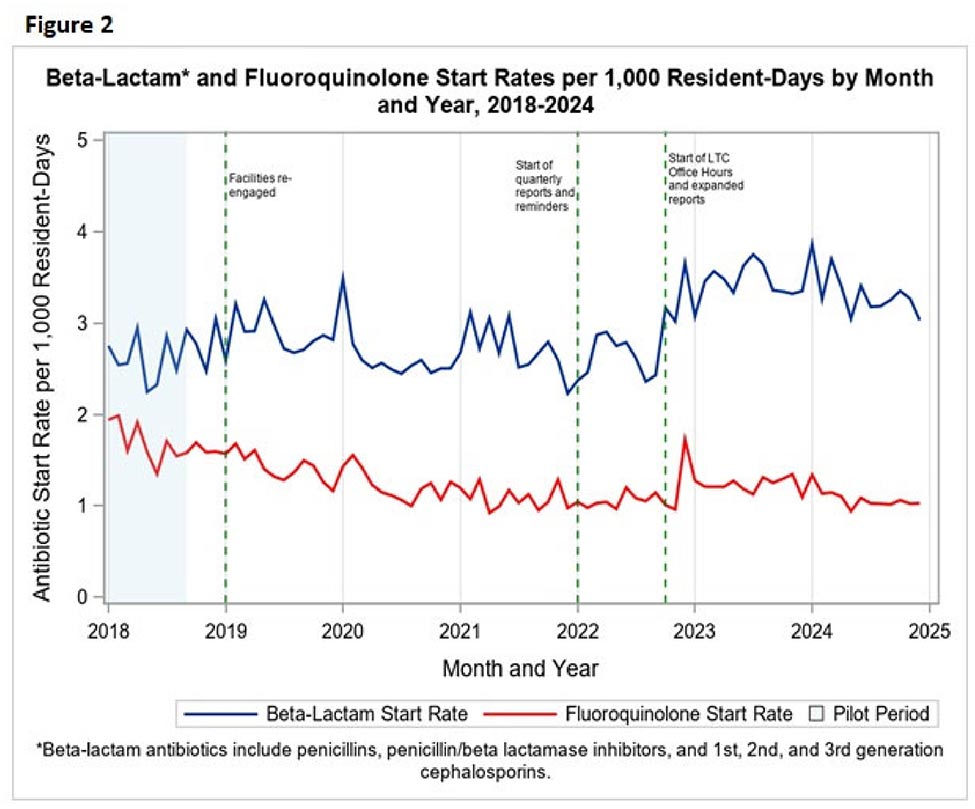
Beta-lactam and fluoroquinolone start rates per 1,000 resident days by month and year, 2018-2024

**Methods:**

We conducted a pre-/post-intervention study from 2018 to 2024 using voluntarily submitted data from Massachusetts LTC facilities. The primary outcome was the monthly AS rate, normalized per 1,000 resident days. Discrete interventions included LTC re-engagement after a hiatus (01/01/2019), quarterly reports to LTC facilities and reminders to submit AS data (01/01/2022), and LTC office hours webinars and an expanded AS report with more granular data (10/01/2022). Interrupted time-series (ITS) analysis assessed changes in AS rates for fluoroquinolones and beta-lactams (penicillins and 1st–3rd generation cephalosporins).

**Results:**

A total of 222 facilities participated and reported at least one month of data. The overall AS rate increased slightly from 7.22 in 2018 to 7.70 in 2024 per 1,000 resident-days (Fig. 1). Fluoroquinolone AS rates declined by 36%, from 1.68 to 1.08 (Fig. 2). In contrast, beta-lactams AS rates increased by 26%, from 2.64 to 3.33. ITS analysis showed a significant increase in beta-lactam prescribing following the implementation of LTC office hours webinars and expanded AS report (*p*< 0.001). No intervention was directly associated with a reduction in fluoroquinolone use.

**Conclusion:**

Public health-led benchmarking demonstrates potential as a scalable strategy for antimicrobial stewardship in LTC settings. The decline in fluoroquinolone use, despite no direct intervention, suggests broader stewardship trends, while increased beta-lactam prescribing reflects the influence of targeted education and reporting. These findings underscore the role of public health departments in advancing data-informed stewardship, especially in resource-limited settings.

**Disclosures:**

All Authors: No reported disclosures

